# Arthroscopic Meniscus Repair: Clinical and Isokinetic Results

**DOI:** 10.1155/DTE.4.119

**Published:** 1998

**Authors:** Christoph Erggelet, Carmen Grosse, Hans-Rudolph Henche, Bart De Koning

**Affiliations:** 1 Orthopaedic Department University of Freiburg Germany; 2 Orthopaedic Department Hospital Rheinfelden Germany; 3 Rehabilitation Center ‘Kurmittelhaus’ Bad Säckingen Germany; 4 Hugstetter Str.55 Freiburg D-79106 Germany

## Abstract

The importance of the menisci for transmitting workloads in the knee joint to protect the articular cartilage is widely acknowledged. Therefore various techniques have been introduced to repair the damaged meniscus.

We performed an arthroscopic meniscus repair with a modified outside-in technique on 29 patients (average 25 years) between 2/91 and 10/94. The average time between trauma and operation was 29 weeks (1–186) – the follow-up 16.3 months (4–49). All the patients were interviewed by phone – 23 were available for clinical respectively isokinetic examination, and categorized following the Lysholm and Lais scores.

Twenty-eight patients were happy with the result of the procedure. Following the Lysholm score we found 78% good/excellent results (Lais score 74%). Isokinetic testing showed a muscular deficit of less than 20% in 91% of the cases for flexion (extension 69%). No significant influence neither of the age of the patient nor the time period between trauma and operation on the outcome of the procedure could be found. No complications were reported.

Based on our results and well aware of the deleterious long term effects of total meniscectomy the arthroscopic menical repair performed by an experienced surgeon should be generous choice of therapy for the treatment of the ruptured meniscus.


*Diagnostic and Therapeutic Endoscopy*, Vol. 4, pp. 119–125
Reprints available directly from the publisher
Photocopying permitted by license only

© 1998 OPA (Overseas Publishers Association)
Amsterdam B.V. Published under license
under the Harwood Academic Publishers imprint,
part of The Gordon and Breach Publishing Group.
Printed in Singapore.

### Arthroscopic Meniscus Repair: Clinical and Isokinetic Results

CHRISTOPH ERGGELET[a,*], CARMEN GROSSE[b], HANS-RUDOLPH HENCHE[b] and BART DE KONING[c]

[a] *Orthopaedic Department, University of Freiburg, Germany;* [b] *Orthopaedic Department, Hospital Rheinfelden, Germany;* [c] *Rehabilitation Center, 'Kurmittelhaus' Bad Säckingen, Germany*

*(Received 4 February 1997; Revised 25 June 1997; In final form 25 August 1997)*

**The importance of the menisci for transmitting workloads in the knee joint to protect the articular cartilage is widely acknowledged. Therefore various techniques have been introduced to repair the damaged meniscus.**

**We performed an arthroscopic meniscus repair with a modified outside-in technique on 29 patients (average 25 years) between 2/91 and 10/94. The average time between trauma and operation was 29 weeks (1–186) – the follow-up 16.3 months (4–49). All the patients were interviewed by phone – 23 were available for clinical respectively isokinetic examination, and categorized following the Lysholm and Lais scores.**

**Twenty-eight patients were happy with the result of the procedure. Following the Lysholm score we found 78% good/excellent results (Lais score 74%). Isokinetic testing showed a muscular deficit of less than 20% in 91% of the cases for flexion (extension 69%). No significant influence neither of the age of the patient nor the time period between trauma and operation on the outcome of the procedure could be found. No complications were reported.**

**Based on our results and well aware of the deleterious long term effects of total meniscectomy the arthroscopic menical repair performed by an experienced surgeon should be generous choice of therapy for the treatment of the ruptured meniscus.**

*Keywords:* Meniscus repair, Isokinetic testing, Arthroscopy, Suture

### INTRODUCTION

The importance of the menisci for transmitting workloads in the knee joint to protect the articular cartilage has been historically underestimated but is now widely acknowledged [1,2]. The deleterious long term effects of total meniscectomy are not only described by Cox [3–6] but McGinty and others [7,8] also emphasized the advantages of partial meniscectomy in light of long term morbidity.

In order to preserve meniscal function many arthrotomic and arthroscopic techniques have been

* Corresponding author. Hugstetter Str.55 D-79106 Freiburg, Germany. Tel.: +49 761 270 2608. Fax: +49 761 270 2675.

developed to repair lesions in the peripheral third of the menisci [9–14] beginning with the pioneer work of Annondale in 1885 [15].

The outcome of arthroscopic meniscus repair can be evaluated either by clinical function using scores [16,17], MRI-scanning [18] or biometrical measures such as isokinetic testing.

We will present clinical and isokinetic results after arthroscopic repair of the ruptured meniscus.

### MATERIALS AND METHODS

Between February 1991 and October 1994 we performed 30 arthroscopic meniscal repairs on 29 patients. They all reported about a distortion trauma of the knee up to 29 months prior to their presentation in our clinic. Historically there was no case of knee pain before the trauma. The clinical findings consisted of unicompartimental knee pain, positive meniscus signs and a 'clicking' in the knee at 'wrong' movements in 13 cases. Concomitant osteoarthritis was ruled out by conventional radiographs. In 5 cases MRI findings indicated a meniscus lesion.

All patients could be interviewed by phone, 23 were available for additional follow-up examination. Patient data is shown in Table I.

The arthroscopy was carried out under standard settings [19]. After inspecting the menisci the indication for the repair was set up by the following criteria as discussed a.o. by Arnotzky and Warren [20]: Bucket-handle ruptures of the anterior and intermediate part of the meniscus near the base line. On the lateral side a suture posterior to the popliteus tendon was avoided. A reconstruction of the ACL was performed at the same time if applicable ($n = 18$).

TABLE I Data of patients available for follow-up examination after arthroscopic meniscus repair

| Patient data | Follow-up examination |
|---|---|
| Number of patients | 23 |
| Sex | |
| Male | 19 |
| Female | 4 |
| Age (years) | 25 (15–45) |
| Operation | |
| Left | 16 |
| Right | 7 |
| Meniscus repair + ACL | 18 |
| Only meniscus repair | 5 |
| Time trauma/operation (weeks) | 29 (1–186) |
| Follow-up (months) | 16 (4–49) |

For the suture itself we selected a modified outside-in technique based on the method described by O'Donnell [21]: Via the loop of a 0–0 PDS thread and one, respectively two adjacent (1 cm) PDS threads, guided by a 16 G needle, a matress suture was set up and tightened subcutaneously. This procedure was repeated if necessary (Figs. 1 and 2).

The post op regime consisted mainly of intensive physical therapy with limitation of flexion at 60° for six weeks with or without bracing, depending on the compliance of the patient. In case of an ACL reconstruction the rehabilitation program followed different guidelines including a four week limitation of weight bearing and bracing for three months. In all cases we recommended to refrain from contact sports for at least six months.

The follow-up assessment included a clinical examination based on the rating scores following Lysholm [16] and Lais [22] as well as on isokinetic testing with a CYBEX 6000. This system including the well tailored software allows the measurement of deficits regarding muscular performance in flexion and extension of the knee joint. The isokinetic testing was categorized 'excellent' if the operated knee performed better than the not-operated knee. If the muscular deficit of the injured knee was less than 20% it was considered to be 'good', more than 20% 'satisfactory' and more than 40% 'fair'.

The evaluation of the data followed the measures of descriptive statistics and the correlation tests according to Spearman and McPherson.

### RESULTS

Twenty-eight out of 29 patients expressed their full satisfaction with the meniscus repair. One patient

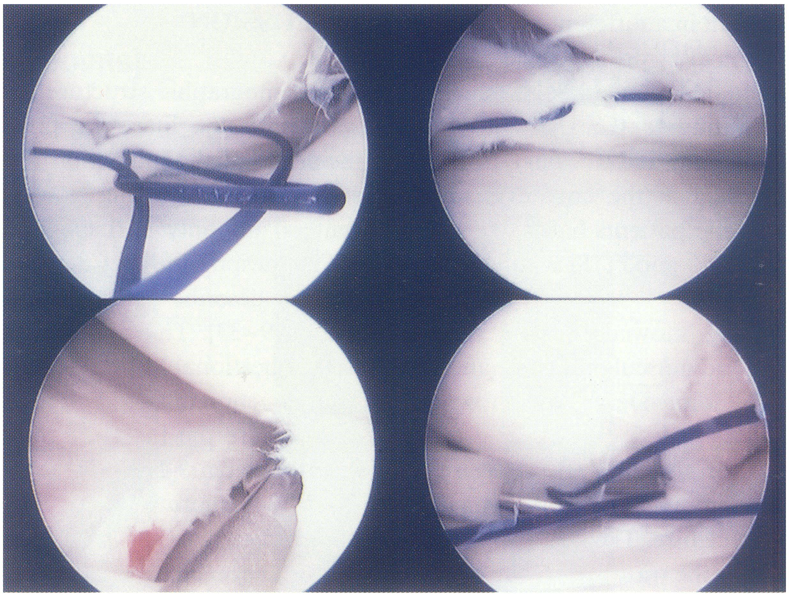

FIGURE 1 Arthroscopic meniscus repair – intraoperative situation. Bottom left: Peripheral lesion of medial meniscus. Bottom right: Positioning of the suture. Top left: Suture before tightening of the central sling. Top right: Result after 2 sutures.

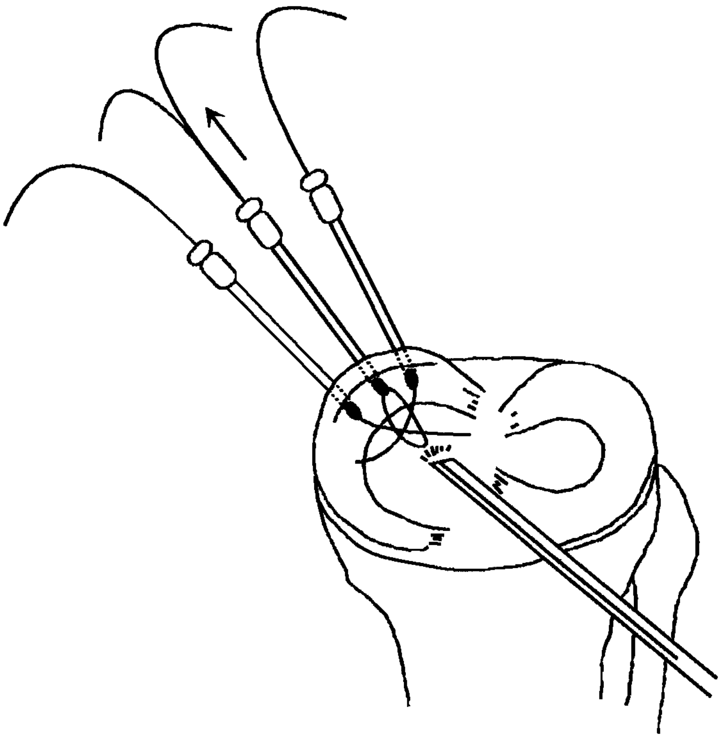

FIGURE 2 Modified outside-in technique for meniscus repair.

had to be operated again in a different hospital for meniscectomy 1.5 years after primary procedure.

Of the 23 reexaminated patients 74% showed excellent results according to the Lysholm-score (4% good, 22% satisfactory and 0% fair). Following the criteria described in the knee evaluation score by Lais 48% of the patients had to be categorized excellent and 30% good (18% satisfactory and 4% fair).

The isokinetic testing showed in 91% of the patients excellent or good muscular performance in the flexion of the knee joint. Details are shown in Fig. 3.

There is no correlation to be found between the age of the patient at the moment of the trauma and the outcome of the procedure. The same applies to the time period between the trauma and the operation (Fig. 4). On the other side there is a significant correlation between the four measures of evaluation. No arthroscopy related complications could be found.

### DISCUSSION

The demographic structure of our patients corresponds to the literature [23–27] as well as the incidence of the indication for meniscal repair. It seems quite surprising that even so called 'knee centers' do only ±10 meniscus repairs per year [28,29] despite the fact that most of the published studies describe a 'success rate' of 80–100% [17,25,29–33]. This appears promising in relation to the questionable long term results after partial or total resection of the meniscus as mentioned above.

What are the factors influencing the results of meniscal repair? The age-factor is touched only by Clark [34] quoting that young tissue heals better than old tissue. The time-factor, which describes the time between trauma and operation, is discussed a.o. by Hamberg [35] indicating that sutures can be successful up to 7 years after trauma. Various authors discussed the indication for repair respectively the localization of the tear/rupture [33,36,37].

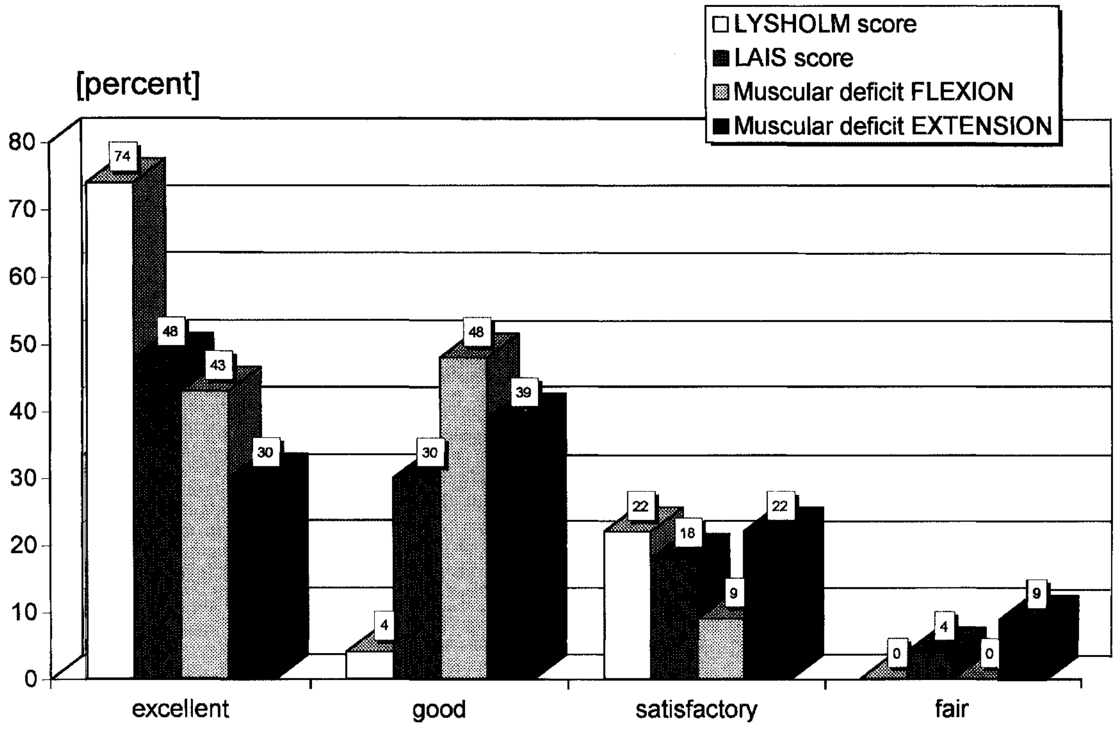

FIGURE 3 Clinical and isokinetic results of arthroscopic meniscus repair categorized in excellent, good, satisfactory and fair.

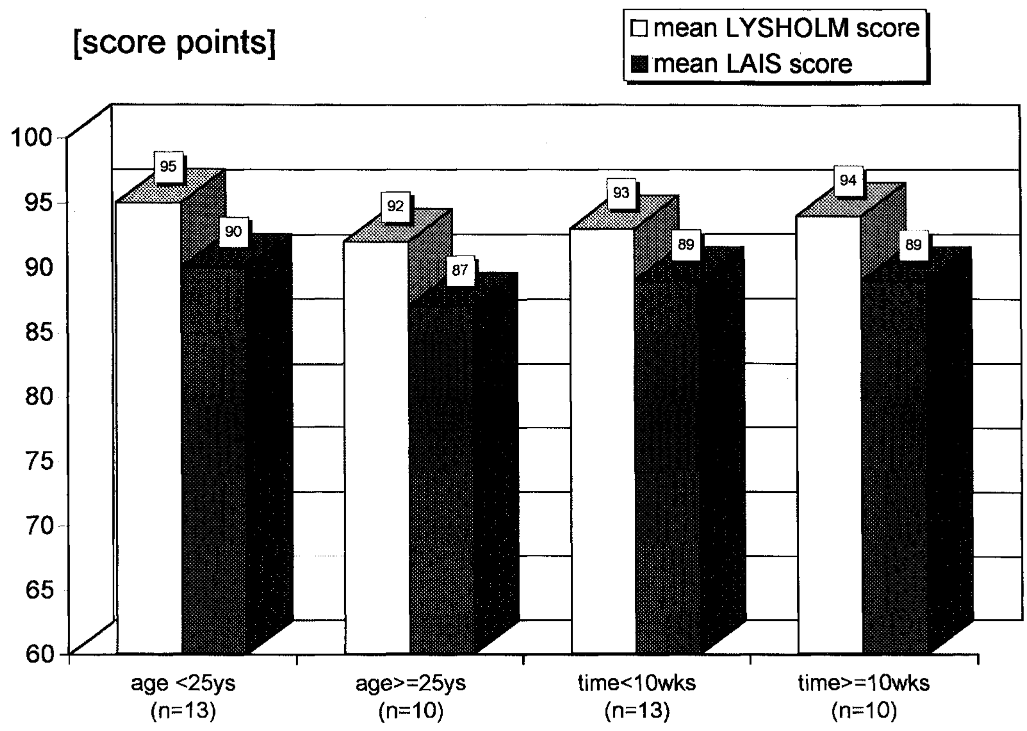

FIGURE 4 Clinical and isokinetic results of arthroscopic meniscus repair regarding the influence of age and time period between trauma and operation of the outcome.

Central lesions have a worse prognostic appearance than peripheral ones [38]. The length of the lesion should not be a criterium – at least not for an experienced surgeon. The biomechanic reactions of the suture itself are described by Kohn and Rössig [37] stating that vertical slings are better than horizontal ones and should be preferred if possible. The terminating knot appears to be weaker (25%) in comparison to the outside-in technique with vertical slings (100%) [37]. This is understandable if you regard the circular construction of the collagen fibres. The clinical importance of the suture intervals seem to be questionable and range between 3 [39], 5 [40] and 10 mm [41]. Resorbable or non-resorbable threads – a robust conclusion cannot be determined in the literature but the tendency goes towards the non-resorbable material [42].

The modified outside-in technique of 3 punctures for 2 sutures used by the author has the advantage of being clinically fast and can be alternated for vertical sutures as well. Costly instrumentation is not necessary.

The rehabilitation regime should be based on the intraarticular situation and the knowledge that an increasing flexion of the knee (more than 60°) stresses the meniscus to a substantial extent [43]. Therefore a dynamic intraoperative examination is essential for the assessment of the stability of the repair. This leads to the postulate of a concomitant treatment of a ACL/PCL lesion with instability [30].

Our results compare well to the relevant literature [17,25,29,31,33] (Table II).

As evaluation measures of meniscal repair MRI is suggested [18] but not (yet) accessible for all the patients. The quality of assessment of the actual status of the reconstructed meniscus is discussed controversially. The very valuable second look arthroscopy has its limits at ethical borders unless the indication results out of persisting or new knee pain.

TABLE II Results of arthroscopic meniscus repair – selected literature

| Author(s) | Year | $N$ | Follow-up (years) | Follow-up rate (%) | Re-ruptures ($N$) | Healing (%) | Evaluation method |
|---|---|---|---|---|---|---|---|
| Strand *et al.* | 1984 | 53 | 2–8 | | 7 | 86 | cl |
| Jacob/Stäubli *et al.* | 1986 | 54 | 3 | | 3 | 89 | ? |
| Rosenberg, T. *et al.* | 1986 | 29 | 0.3 | | — | 100 | cl/s |
| Stone, R.G. | 1990 | 31 | 4.1 | 33 | ? | 88 | cl/sc |
| Funke, E. *et al.* | 1993 | 41 | 43 | 95 | 6 | 93 | cl |
| Hackenbruch, W. | 1993 | 51 | 2.5 | | 1 | 98 | cl |
| Jensen, N.C. *et al.* | 1994 | 49 | 1–6.3 | | 11 | | cl,sc |

cl = clinical examination, s = arthroscopic examination, sc = score evaluation.

Isokinetic testing is not very commonly described but reflects well one quality of an operative result: the clinical function of a joint. The other quality is at least as important but inaccessible to 'statistical treatment' and comparison: the well-being in an every day environment.

The CYBEX 6000 system quantifies a.o. the muscular performance of extension and flexion in the knee joint. The flexion power seems to raise faster from post-operative lethargy than extension (91% vs 69% excellent/good). The reasons for those findings might be sourced in persistent pain ('no training with pain') or the post-op restrictions after ACL-reconstruction.

Conclusively the indication for meniscal repair should be set generously regarding the good to excellent results. The outside-in technique with or without modifications, vertical slings and non-resorbable sutures offer a good clinical performance and can be/should be carried out arthroscopically. Patients' age and the period of time between trauma and operation seem to be of neglectable importance. A concomitant ACL/PCL lesion should be treated simultaneously. The rehabilitation regime is dependent on the intra-op situation – limitation of flexion for 6 weeks is recommended.

### References

[1] Walker, P. and Erkmann, M. The role of menisci in force transmission across the knee. *Clin. Orthop.* 1975; **109**: 184–92.

[2] Krause, M. and Pope, M. Mechanical changes in the knee after meniscectomy. *J. Bone Joint Surg.* [*Am*] 1976; **5**: 599–604.

[3] Cox, J.S., Nye, C.E., Schaefer, W.W. and Woodstein, I.J. The degenerative effects of partial and total resection of the medial meniscus in dogs' knees. *Clin. Orthop.* 1975; **109**: 178–83.

[4] Tapper, E.M. and Hoover, N.W. Late results after meniscectomy. *J. Bone Joint Surg.* [*Am*] 1969; **51**(3): 517–26: assim.

[5] Huckell, J. Is meniscectomy a benign procedure? A long term follow up study. *Can. J. Surg.* 1965; **8**: 254–60.

[6] Johnson, R.J., Kettelkamp, D.B., Clark, W. and Leaverton, P. Factors effecting late results after meniscectomy. *J. Bone Joint Surg.* [*Am*] 1974; **56**(4): 719–29.

[7] McGinity, J.B., Geuss, L.F. and Marvin, R.A. Partial or total meniscectomy: a comparative analysis. *J. Bone Joint Surg.* [*Am*] 1977; **59**(6): 763–6.

[8] Northmore-Ball, M., Dandy, D. and Jackson, R. Arthroscopic open partial and total meniscectomy: a comparative study. *J. Bone Joint Surg.* [*Br*] 1983; **65**: 400–4.

[9] Morgan, C.D., Wojtys, E.M., Casscells, C.D. and Casscells, S.W. Arthroscopic meniscal repair evaluated by second-look arthroscopy. *Am. J. Sports Med.* 1991; **19**(6): 632–7; discussion 637–8.

[10] Morgan, C.D. and Casscells, S.W. Arthroscopic meniscus repair: a safe approach to the posterior horns. *Arthroscopy* 1986; **2**(1): 3–12.

[11] Morgan, C.D. The "all-inside" meniscus repair. *Arthroscopy* 1991; **7**(1): 120–5.

[12] Landsiedl, F. Improved outside-in technique of arthroscopic meniscal suture. *Arthroscopy* 1992; **8**(1): 130–1.

[13] Henning, C.E., Clark, J.R., Lynch, M.A., Stallbaumer, R., Yearout, K.M. and Vequist, S.W. Arthroscopic meniscus repair with a posterior incision. *Instr. Course Lect.* 1988; **37**: 209–21.

[14] Rosenberg, T.D., Paulos, L.E., Wnorowski, D.C. and Gurley, W.D. Arthroscopic surgery: meniscus refixation and meniscus healing [Arthroskopische Chirurgie: Meniskusrefixation und Meniskusheilung]. *Orthopade* 1990; **19**(2): 82–9.

[15] Annondale, T. An operation for displaced semilunar cartilage. *BMJ* 1885; **1**: 779.

[16] Lysholm, J. and Gillquist, J. Evaluation of knee ligament surgery results with special emphasis on use of a scoring scale. *Am. J. Sports Med.* 1982; **10**(3): 150–4.

[17] Stone, R.G., Frewin, P.R. and Gonzales, S. Long-term assessment of arthroscopic meniscus repair: a two- to six-year follow-up study. *Arthroscopy* 1990; **6**(2): 73–8.

[18] Castro, W.H., Jerosch, J. and Assheuer, J. Value of computerized tomography and nuclear magnetic resonance tomography in preoperative diagnosis of meniscus lesions and ligamentous lesions of the knee joint [Der Aussagewert der Computertomographie und Kernspintomographie bei der praoperativen Diagnostik von Meniscuslasionen und Bandlasionen des Kniegelenks]. *Chirurg.* 1991; **62**(5): 394–8.

[19] Henche, H.R. Vorbereiten des Patienten für die Arthroskopie. In: Henche, H.R. and Holder, J. (eds). *Die Arthroskopie des Kniegelenkes*. Second Edition. Springer, Berlin, Heidelberg, New York, 1987; **6**: 25–8.

[20] Arnoczky, S.P. and Warren, R.F. The microvasculature of the human meniscus. *Am. J. Sports Med.* 1982; **10**: 90–5.

[21] O'Donnell, J.B., Ruland, C.M. and Ruland, L.J. A modified outside-in meniscal repair technique. *Arthroscopy* 1993; **9**(4): 472–4.

[22] Lais, M., Jundt, W., Huber, D. and Henkemeyer, H. Vergleichende Ergebnisse verschiedener Behandlungsverfahren bei vorderen Kreuzbandrupturen. *Praktische Sport-traumatologie und Sportmedizin* 1989; **4**: 16–7.

[23] Buseck, M.S. and Noyes, F.R. Arthroscopic evaluation of meniscal repairs after anterior cruciate ligament reconstruction and immediate motion. *Am. J. Sports Med.* 1991; **19**(5): 489–94.

[24] Hanks, G.A., Gause, T.M., Sebastianelli, W.J., O'Donnell, C.S. and Kalenak, A. Repair of peripheral meniscal tears: open versus arthroscopic technique. *Arthroscopy* 1991; **7**(1): 72–7.

[25] Jakob, R.P., Staubli, H.U., Zuber, K. and Esser, M. The arthroscopic meniscal repair: techniques and clinical experience. *Am. J. Sports Med.* 1988; **16**(2): 137–42.

[26] Messner-Sommerlath, K. Reattachment of the meniscus: techniques, long-term results and recommendation for individual treatment [Die Meniskusrefixation. Techniken, Langzeitergebnisse und Empfehlung zur individuellen Behandlung]. *Orthopade* 1994; **23**(2): 137–42.

[27] Miller, D.B., Jr. Arthroscopic meniscus repair. *Am. J. Sports Med.* 1988; **16**(4): 315–20.

[28] Ryu, R.K. and Dunbar, W.H. Arthroscopic meniscal repair with two-year follow-up: a clinical review. *Arthroscopy* 1988; **4**(3): 168–73.

[29] Jensen, N.C., Riis, J., Robertsen, K. and Holm, A.R. Arthroscopic repair of the ruptured meniscus: one to 6.3 years follow up. *Arthroscopy* 1994; **10**(2): 211–4.

[30] Rosenberg, T.D., Scott, S.M., Coward, D.B., Dunbar, W.H., Ewing, J.W. and Johnson, C.L. *et al.* Arthroscopic meniscal repair evaluated with repeat arthroscopy. *Arthroscopy* 1986; **2**(1): 14–20.

[31] Strand, T., Engesaeter, L.B. and Molster, A.O. Meniscus repair in knee ligament injuries. *Acta Orthop. Scand.* 1984; **56**(2): 130.

[32] Funke, E., Marty, M., Munzinger, U. and Drobny, T. Ergebnisse der arthroskopischen Meniskusnaht. *Arthroskopie* 1993; **6**: 76–9.

[33] Hackenbruch, W. Arthroskopische Meniskusrefixation. *Arthroskopie* 1993; **6**: 67–72.

[34] Clark, C.R. and Ogden, J.A. Developement of the menisci of the human knee joint. *J. Bone Joint Surg.* [*Am*] 1983; **65**: 538–42.

[35] Hamberg, P., Gillquist, J. and Lysholm, J. Suture of new and old peripheral meniscus tears. *J. Bone Joint Surg.* [*Am*] 1983; **65**(2): 193–7.

[36] Benedetto, K.P., Glotzer, W., Kunzel, K.H. and Gaber, O. The vascularization of the menisci. morphological basis for the repair [Die Gefassversorgung der Menisken Morphologische Grundlagen fur die Refixation]. *Acta Anat.* (*Basel*) 1985; **124**(1–2): 88–92.

[37] Kohn, D. and Rössig, S. Meniskusnaht. *Arthroskopie* 1993; **6**: 63–6.

[38] Rödecker, K. and Nagelschmidt, M. Aufbau und Heilungsvermögen des Meniskus. *Arthroskopie* 1993; **6**: 56–62.

[39] Cannon, W.D. Arthroscopic meniscus repair. In: McGinty, J. (eds). *Operative arthroscopy*. Raven Press, New York, 1991: 237–251.

[40] Wirth, C.J., Rodriguez, M. and Milachowski, K. Meniskusnaht–Meniskusersatz. Thieme. Stuttgart, New York: 1988.

[41] Jakob, R.P., Ballmer, P.M. and Zuber, K. *et al.* Meniskusrefixation unter besonderer Berüksichtigung der arthroskopischen Technik. In: Jakob, R.P., Stäubli, H.U. (eds). *Kniegelenk und Kreuzbänder*. Springer, Berlin, Heidelberg, New York, 1990: 339–49.

[42] DeHaven, K.E. Meniscectomy versus repair: clinical experience. In: Mow, V.S., Arnoczky, S.P. and Jackson, D.W. (eds). Knee meniscus: basic and clinical foundations. Raven Press, New York, 1992: 131–9.

[43] Ruetsch, H. and Morscher, E. Measurement of the rotatory instability of the knee joint. In: Chapchal, G. (eds.). Injuries of the ligaments and their repair. Thieme, Stuttgart, New York, 1977: 116–22.

